# A Rare Case of Serratia marcescens Causing Mycotic Aneurysm and Septic Emboli in Intravenous Drug User

**DOI:** 10.7759/cureus.9419

**Published:** 2020-07-27

**Authors:** Kyaw M Hlaing, Salem Gaballa, Jasmine Saini, Dave Fintel, Kashyap Patel

**Affiliations:** 1 Internal Medicine, LewisGale Medical Center, Salem, USA; 2 Medicine, Edward Via College of Osteopathic Medicine, Blacksburg, USA

**Keywords:** mycotic aneurysm, serratia marcescens, ivdu, marfan disease

## Abstract

There are few literatures highlighting the presence of a mycotic aneurysm in the setting of bloodstream infection by Serratia. A 33-year-old male with a history of Marfan syndrome, mitral valve prolapse, and intravenous drug use (IVDU) presented to the ED with fever, nausea, and non-bloody emesis, and vague abdominal pain with concern for sepsis. With the strong association between IVDU and infective endocarditis, transthoracic and transesophageal echocardiograms were performed and were negative for vegetations. Abdominal CT and positron emission tomography (PET) scan were performed and revealed thrombosis at the first jejunal branch of the superior mesenteric artery (SMA), left renal pole infarct, and superior splenic pole infarct. Following CT angiography for potential thrombolysis, aneurysmal formation was discovered proximal to the filling defect within mid-SMA. Blood cultures drawn at presentation grew Serratia marcescens. The patient was treated with appropriate antibiotic, and recommended indefinite anticoagulation. The patient was then recommended to follow up with vascular surgery within two weeks for repeat abdominal CT angiogram.

## Introduction

Serratia marcescens is a facultative anaerobic gram-negative motile bacillus most notably recognized for producing reddish pigment upon colonization. It can be isolated from soil, plants, various water sources, both natural and municipal, and sometimes, human intestinal flora [1]. Historically, community-acquired bloodstream infections of Serratia have been almost exclusively found in intravenous drug users (IVDU). However, nosocomial infections are also common and have been attributed to invasive interventions, such as surgery, bronchoscopy, and foreign body placement. Mycotic aneurysms are dilations of an artery due to damage of the vessel wall by infection. Visceral mycotic aneurysms only comprise 0.1%-0.2% of aneurysms, though the true incidence is unknown [2]. There are few cases reporting the unique presentation of Serratia bloodstream infection and mycotic aneurysm formation occurring in IVDU [1]. This report discusses a case of sepsis and visceral mycotic aneurysm discovery in IVDU with a history of mitral valve prolapse (MVP), in the absence of discoverable infective endocarditis (IE).

## Case presentation

A 33-year-old male patient with a history of Marfan syndrome, MVP, IVDU, and chronic hepatitis C presented with high fevers, migraines, severe nausea, and non-bloody emesis for several days prior to admission. He also reported associated headaches, mild abdominal pain, and changes in vision. The patient reported family history of Marfan syndrome in his biological father. His opioid dependence (cocaine and morphine IV) had been treated with buprenorphine/naloxone for three years, but he admitted to recent relapse of IVDU one week prior.

The vital signs on admission were remarkable for temperature of 102˚F, heart rate of 120 beats/minute, and blood pressure of 93/52 mmHg. Laboratory data were significant for white blood cell count (WBC) of 15.75 x 10^9^/L, troponin (cT) of 0.187 ng/mL, creatinine (Cr) 0.97 mg/dL, and lactic acid of 2.25 mmol/L (Table 1). On physical examination, the patient appeared cachectic and frail, with marfanoid habitus, and pronounced pectus excavatum; his abdomen was diffusely tender with some guarding without rebound tenderness; and a 2/6 systolic murmur over the cardiac apex was noted on auscultation.

**Table 1:** Laboratory values at admission BUN, blood urea nitrogen; ESR, erythrocyte sedimentation rate

Tests	Result	Reference Range
Hemoglobin	10.9 g/dL	14-16 g/dL
Hematocrit	33.7%	40%-52%
White cell count	15.75 x 10^9^/L	4-10 x 10^9^/L
Platelet count	382 x 10^9^/L	150-400 x 10^9^/L
Na	136 mEq/L	135-145 mEq/L
K	4.2 mEq/L	3.5-5.2 mEq/L
Cl	105 mEq/L	96-106 mEq/L
CO_2_	26 mEq/L	23-29 mEq/L
BUN	19 mg/dL	6-20 mg/dL
Creatinine	0.97 mg/dL	0.8-1.2 mg/dL
Albumin	2.8 g/dL	3.4-5.4 g/dL
ESR	30 mm/hr	0-26 mm/hr
Procalcitonin	0.83 ng/mL	0.10-0.25 ng/mL
Troponin	0.187 ng/mL	0-0.045 ng/mL

**Figure 1:**
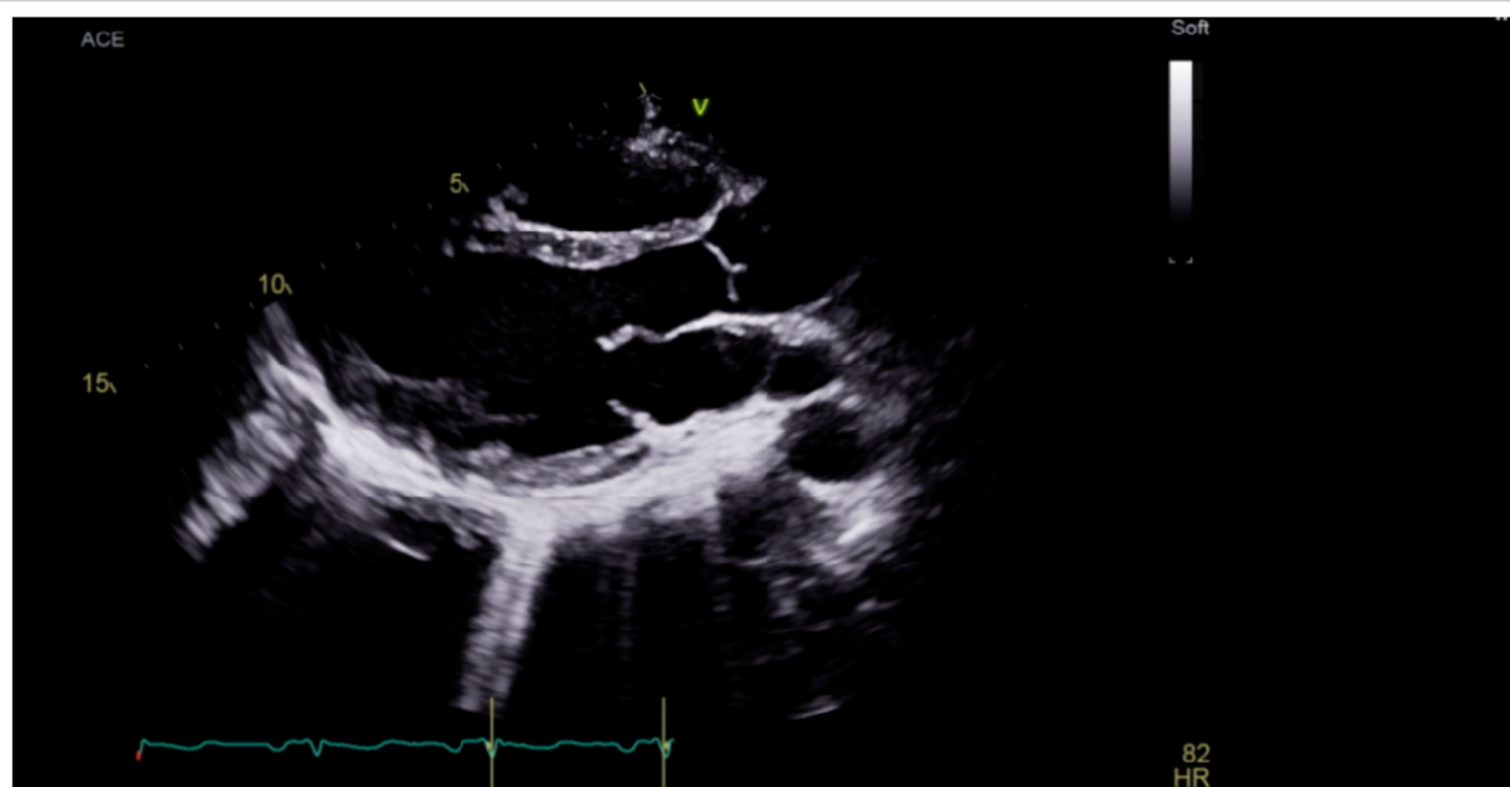
Transthoracic echocardiogram showing no vegetation

**Figure 2:**
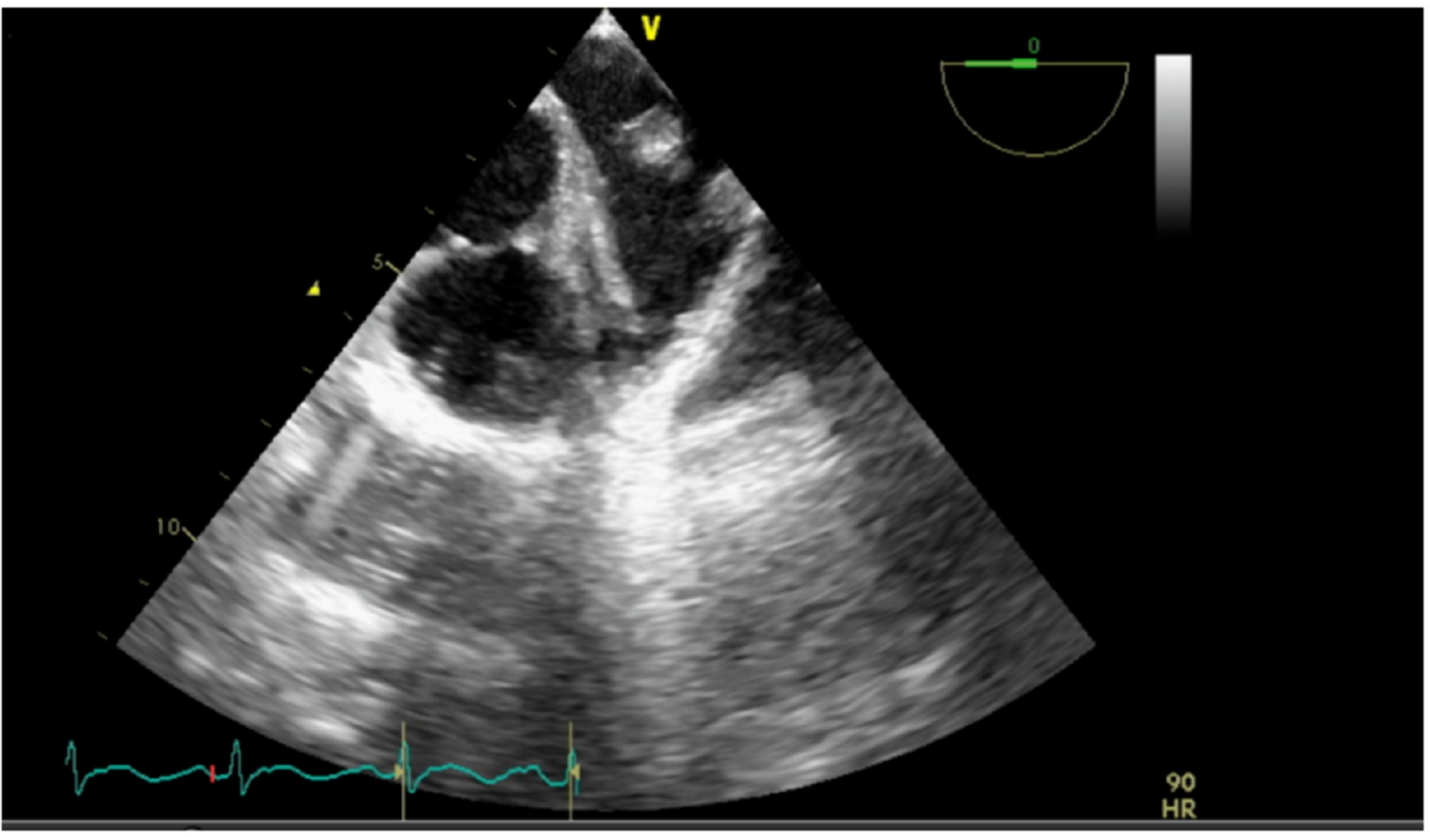
Transesophageal echocardiogram without vegetation

The patient was treated with fluid resuscitation with normal saline bolus (30 mL/kg/hr), maintenance fluid 150 mL/hr, and empiric antibiotic therapy with IV piperacillin/tazobactam and vancomycin that were administered as per sepsis protocol. This patient’s history of IVDU and MVP raised concerns for IE; therefore, transthoracic and transesophageal echocardiograms (TTE and TEE, respectively) were performed that showed no signs of valvular vegetation (Figures 1, 2). Initial blood cultures resulted in four out of four sets growing gram-negative bacillus, further identified as Serratia marcescens. Empiric antibiotics therapy changed to ceftriaxone 1 g IV every 24 hours and metronidazole 500 mg PO every eight hours that were started for gram-negative coverage.

**Figure 3:**
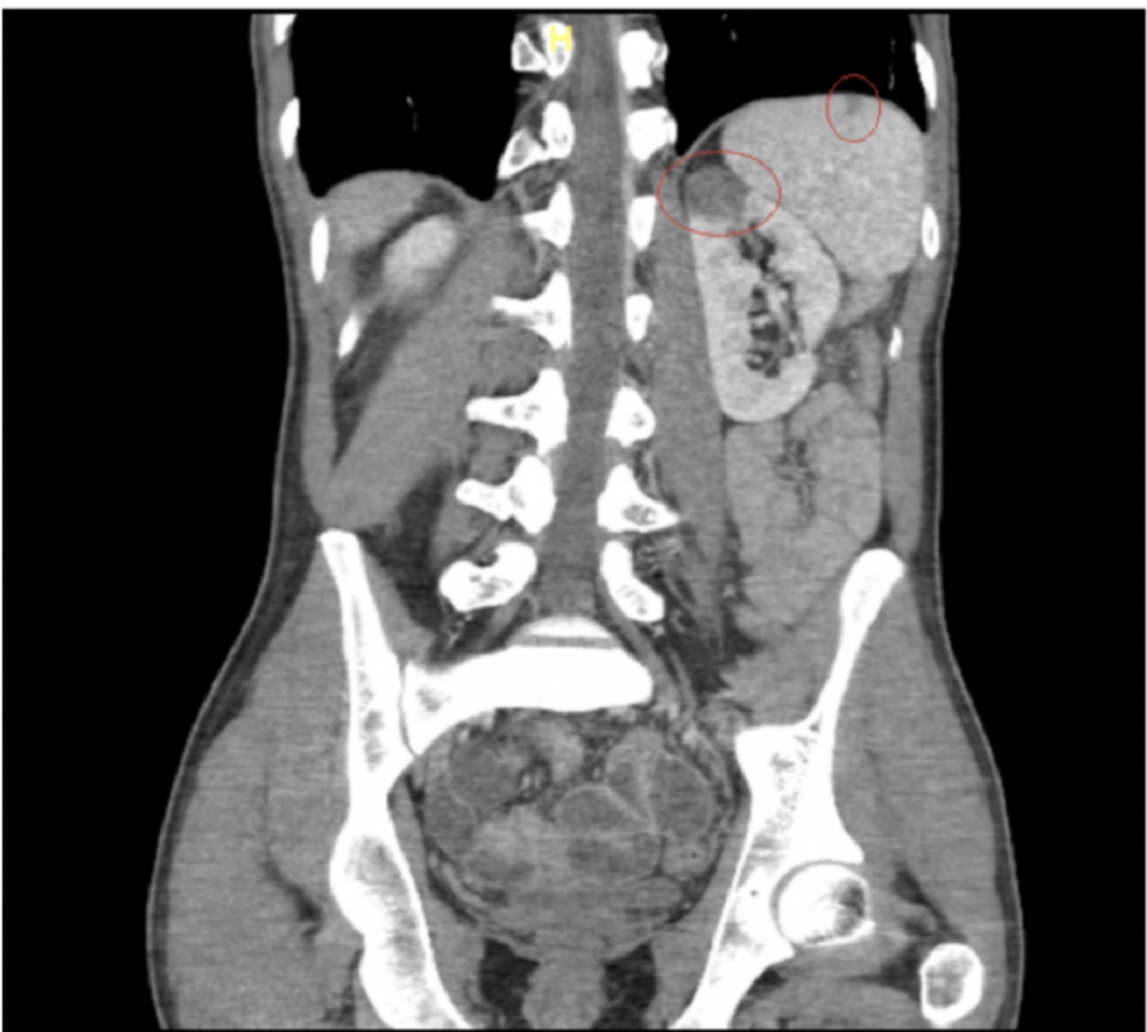
Superior splenic infarct and focal infarct in the left upper renal pole

**Figure 4:**
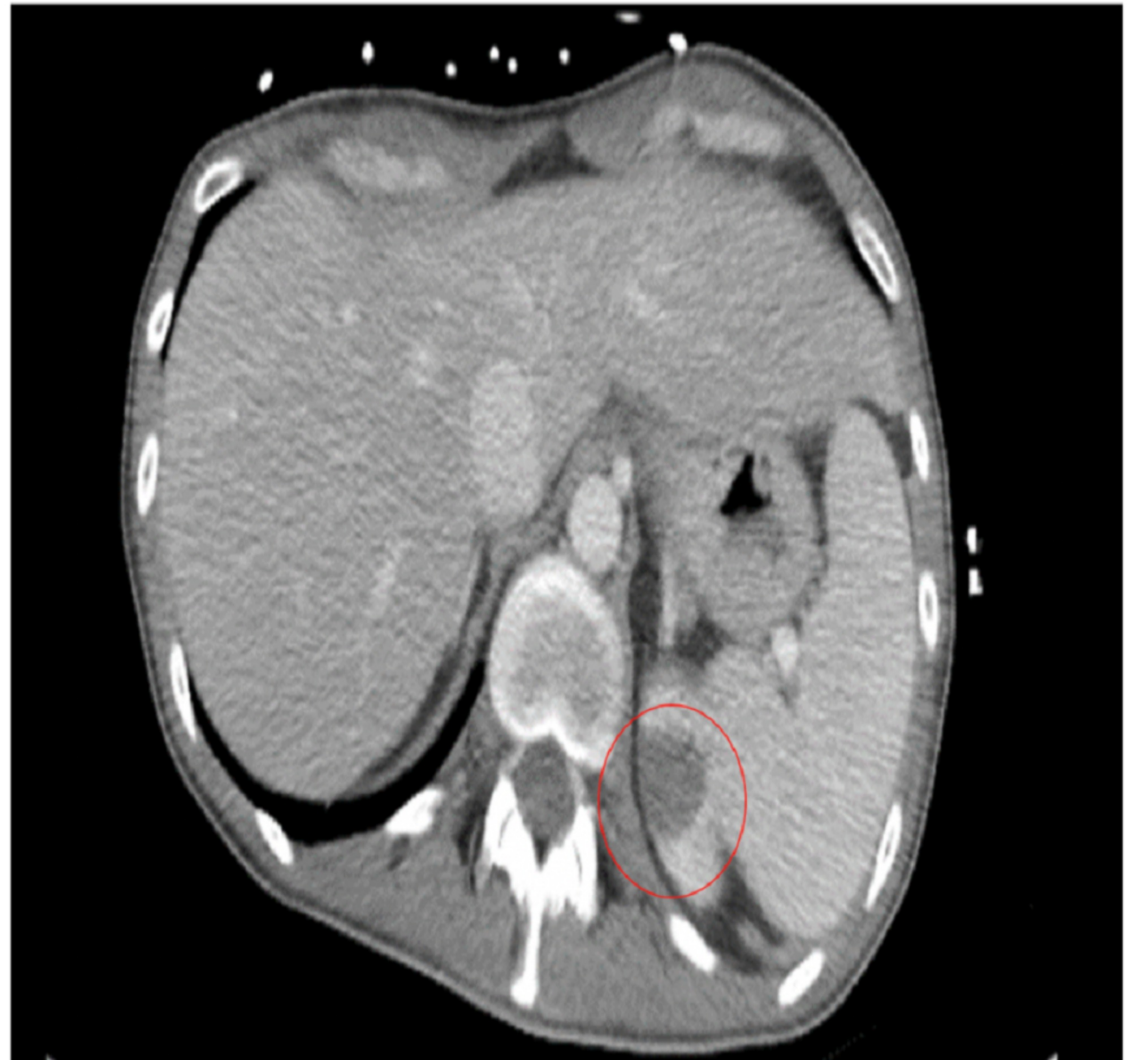
Left renal infarction

On day 3, the patient began complaining of progressive, diffuse abdominal pain, and continued non-bloody emesis. CT of the abdomen/pelvis revealed non-specific bowel gas patterns with a small splenic infarct and area of focal infarct in the left upper renal pole (Figures 3, 4). IV heparin was initiated to prevent further infarctions.

**Figure 5:**
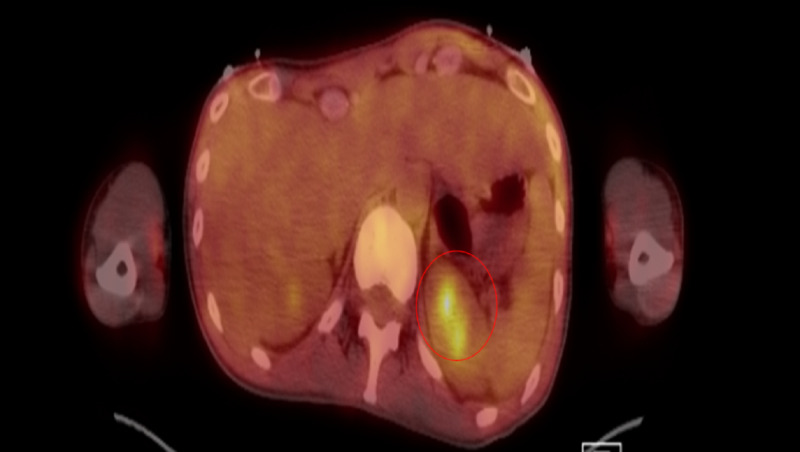
Infection positron emission tomography scan showing increased uptake in the left upper renal pole

**Figure 6:**
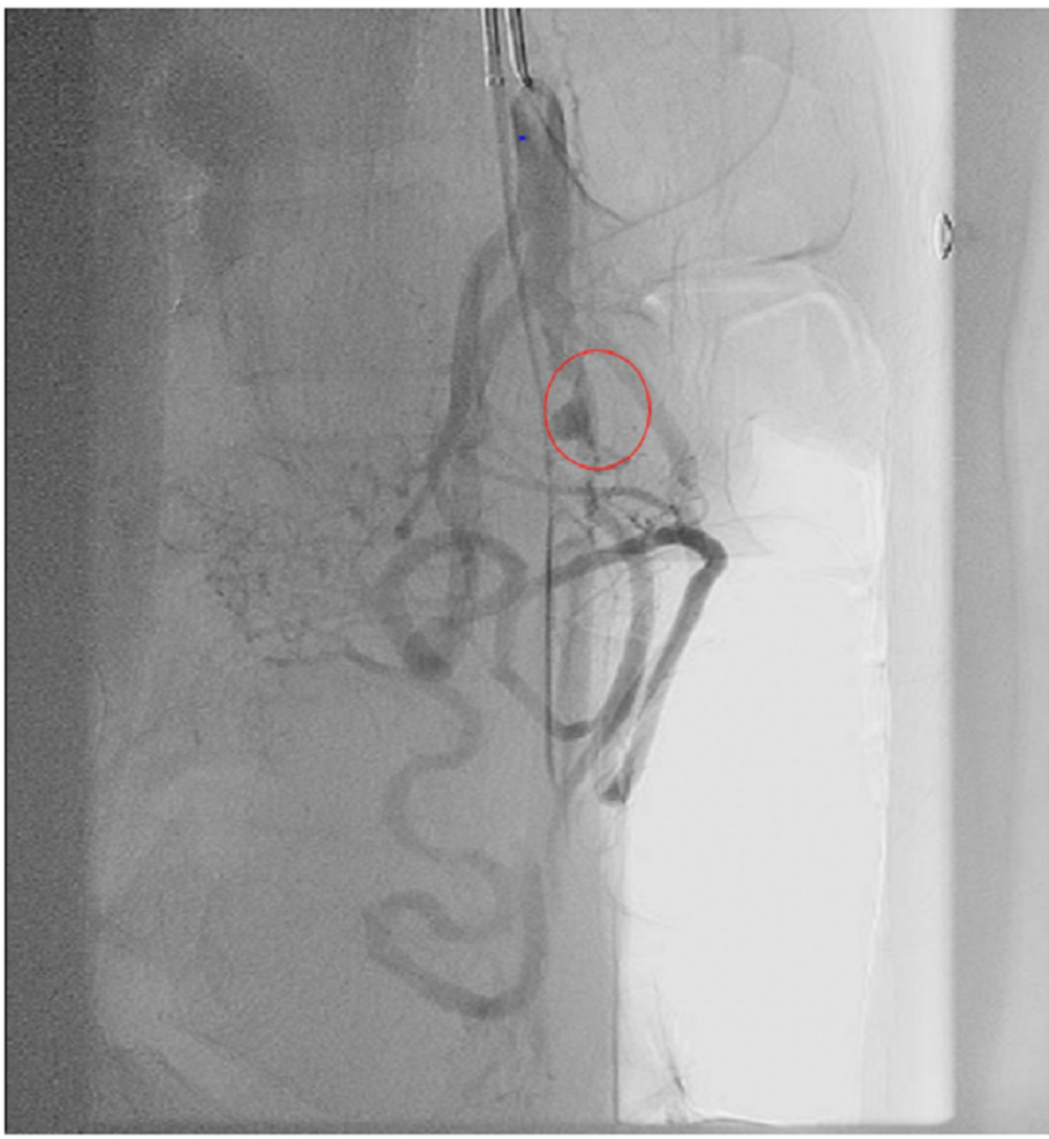
Aneurysmal formation proximal to filling defect

Drug sensitivities showed organism susceptibility to trimethoprim/sulfamethoxazole (TMP-SMX), and therefore, ceftriaxone was substituted with TMP-SMX 160/800 mg PO BID and continued metronidazole therapy. The patient starting to show clinical improvement and WBCs continued to trend down. Positron emission tomography (PET) scan was performed to evaluate the source of infection, and revealed an area of thrombosis at the first jejunal branch of the superior mesenteric artery (SMA), as well as increased uptake at the left renal pole (Figure 5). Interventional radiology was consulted for possible catheter-directed thrombolysis and CT angiogram (CTA). Unexpectedly, a large, obstructive distal saccular aneurysmal formation was discovered proximal to the mid-SMA filling defect (Figure 6).

Vascular surgery was consulted for further management of mycotic aneurysm, and the patient was deemed to be a poor surgical candidate due to the distal nature of aneurysm. Therefore, a conservative management was recommended with long-term anticoagulant therapy. The patient was discharged with apixaban 5 mg twice a day with two-week course of TMP/SMX and metronidazole TID orally. The patient was then recommended to follow up with primary care physician and vascular surgeon with repeat CTA in two weeks. Unfortunately, the patient lost follow-up as recommended and the contact was unsuccessful. 

## Discussion

Global prevalence of IVDU increases, as does presentation of unique infections by unlikely sources such as Serratia spp. It is important to first consider the risk factors at play when trying to understand the etiology of the patient’s disease. IVDU can introduce a myriad of flora to the human body by way of contaminated needles, and while uncommon, Serratia marcescens is one of the possible organisms. It is suggested that the ultimate source of inoculation of this bacterium may have occurred in exposure of drug paraphernalia to contaminated water sources. However, ‘needle licking’ prior to injection in IVDU may be an underappreciated mechanism for infection, as the organism may exist as part of the normal intestinal flora [3].

Marfan syndrome is another major risk factor that may have contributed to aneurysmal development in this patient, as it may weaken the tissue and changes elasticity within the vessels [4]. Aneurysms are characterized by vessel dilations and are categorized as mycotic aneurysms when they are caused by infection. Mycotic aneurysms can develop from adjacent inoculation, septic emboli, vascular trauma, or direct infectious invasion from contaminated needle. With a history of IVDU and pre-existing valvular abnormality, septic emboli from IE would seem a likely etiological source of mycotic aneurysm for this patient. Serratia marcescens adheres much less readily to the valvular endothelium as compared to the gram-positive bacteria typically associated with IE [1]. It is a possibility that there were vegetations for a transient period, or that the vegetation was too small to be detected by echocardiogram. The latter may be an unlikely scenario as TEE is highly sensitive (ranging from 90% to 100% sensitivity) for detecting valvular vegetations [5].

The clinical presentation of SMA aneurysms can be abdominal pain, non-bloody emesis, and/or persistent bacteremia. Blood cultures and CTA remain the gold standard for diagnosis of SMA mycotic aneurysm. Further work-up with TTE and TEE was recommended to explore the source. It is noted in the literature that SMA mycotic aneurysms have an estimated risk of rupture of 50%, and higher mortality rates [2]. Additionally, Serratia marcescens may cause more virulent infection and increased risk of rupture [6]. With both visceral mycotic aneurysm and gram-negative infection, the patient was determined to be at high risk of rupture and poor prognosis. 

In regard to treatment, effective antibiotic therapy guidelines for Serratia are not well defined. It is shown that the use of aminoglycosides, fourth-generation cephalosporins, monobactams, carbapenems, and fluoroquinolones have higher success rate. Resistance against ampicillin and first-, second-, and third-generation cephalosporins have been recorded, as well as identification of recent imipenem-resistant strains. In addition to antibiotic therapy, the mycotic aneurysm requires surgical repair if amendable [3]. After surgical repair, the patient need to follow up with CTA for the resolution of mycotic aneurysm. The patient was determined to be a poor surgical candidate with multiple comorbidities, due to the distal nature of aneurysm; therefore, a conservative therapy with an indefinite anticoagulant and follow-up imaging is recommended. There is no literature about the prefer anticoagulant between vitamin K antagonist and newer oral anticoagulants for better compliance and effectiveness. Untreated mycotic aneurysm has poor outcome with persistent bacteremia, rupture, and serious complication [7,8]. Therefore, we choose apixaban for indefinite therapy.

## Conclusions

Serratia marcescens is a rare cause of mycotic aneurysm and IE. CTA and blood cultures remain the gold standard for diagnosis. The usual management is surgical repair in addition to the antibiotic therapy. For the non-surgical candidates, lifelong anticoagulant therapy is recommended as alternative with follow-up imaging. Multidisciplinary care for mycotic aneurysm is recommended for better outcomes.
